# TCGA Expedition: A Data Acquisition and Management System for TCGA Data

**DOI:** 10.1371/journal.pone.0165395

**Published:** 2016-10-27

**Authors:** Uma R. Chandran, Olga P. Medvedeva, M. Michael Barmada, Philip D. Blood, Anish Chakka, Soumya Luthra, Antonio Ferreira, Kim F. Wong, Adrian V. Lee, Zhihui Zhang, Robert Budden, J. Ray Scott, Annerose Berndt, Jeremy M. Berg, Rebecca S. Jacobson

**Affiliations:** 1 Department of Biomedical Informatics, University of Pittsburgh School of Medicine, Pittsburgh, PA, United States of America; 2 University of Pittsburgh Cancer Institute, Pittsburgh, PA, United States of America; 3 Department of Human Genetics, University of Pittsburgh School of Public Health, Pittsburgh, PA, United States of America; 4 Center for Simulation and Modeling, University of Pittsburgh, Pittsburgh, PA, United States of America; 5 Pittsburgh Supercomputing Center, Carnegie Mellon University, Pittsburgh, PA, United States of America; 6 Department of Pharmacology and Cell Biology, University of Pittsburgh, Pittsburgh, PA, United States of America; 7 Magee-Women’s Research Institute, Pittsburgh, PA, United States of America; 8 UPMC Corporate Services, Pittsburgh, PA, United States of America; 9 Institute for Precision Medicine, University of Pittsburgh, Pittsburgh, PA, United States of America; Flinders University, AUSTRALIA

## Abstract

**Background:**

The Cancer Genome Atlas Project (TCGA) is a National Cancer Institute effort to profile at least 500 cases of 20 different tumor types using genomic platforms and to make these data, both raw and processed, available to all researchers. TCGA data are currently over 1.2 Petabyte in size and include whole genome sequence (WGS), whole exome sequence, methylation, RNA expression, proteomic, and clinical datasets. Publicly accessible TCGA data are released through public portals, but many challenges exist in navigating and using data obtained from these sites. We developed TCGA Expedition to support the research community focused on computational methods for cancer research. Data obtained, versioned, and archived using TCGA Expedition supports command line access at high-performance computing facilities as well as some functionality with third party tools. For a subset of TCGA data collected at University of Pittsburgh, we also re-associate TCGA data with de-identified data from the electronic health records. Here we describe the software as well as the architecture of our repository, methods for loading of TCGA data to multiple platforms, and security and regulatory controls that conform to federal best practices.

**Results:**

TCGA Expedition software consists of a set of scripts written in Bash, Python and Java that download, extract, harmonize, version and store all TCGA data and metadata. The software generates a versioned, participant- and sample-centered, local TCGA data directory with metadata structures that directly reference the local data files as well as the original data files. The software supports flexible searches of the data via a web portal, user-centric data tracking tools, and data provenance tools. Using this software, we created a collaborative repository, the Pittsburgh Genome Resource Repository (PGRR) that enabled investigators at our institution to work with all TCGA data formats, and to interrogate these data with analysis pipelines, and associated tools. WGS data are especially challenging for individual investigators to use, due to issues with downloading, storage, and processing; having locally accessible WGS BAM files has proven invaluable.

**Conclusion:**

Our open-source, freely available TCGA Expedition software can be used to create a local collaborative infrastructure for acquiring, managing, and analyzing TCGA data and other large public datasets.

## Background

Studies interrogating the landscape of genomic alterations in disease are becoming increasingly complex, producing massive data sets derived from next generation sequencing (NGS) platforms [1, 2].One such project is The Cancer Genome Atlas Project (TCGA) [3], a National Cancer Institute (NCI)-funded consortium project whose objective is to profile at least 500 cases of 20 different tumor types using genomic platforms and to make these data, both raw and processed, available to all researchers. TCGA data are currently over 1.2 Petabyte in size and include whole genome sequence (WGS), whole exome sequence (WXS), methylation, RNA expression, proteomic, and clinical datasets. Broad access to this dataset is intended to allow independent research groups to simultaneously interrogate the data and accelerate the discovery of biomarkers associated with cancer initiation, progression and response to therapy. Over 200 publications have already resulted from TCGA data, including the discovery of previously unknown molecular subtypes of cancers [4–14], characterization of mutational loads across different cancers [15], role of miRNAs in cancers [16–18], and relationships between methylation, mutation, gene expression, and clinical phenotypes [4, 8, 12, 16, 19].

TCGA data consist of both protected and publicly available data; the former include sequencing data and files containing germline variants while the latter are raw files from platforms that do not produce sequence data, and processed files from all platforms with the exception of germline variants; for example, publicly available data include gene expression values and somatic variants for each sample. Publicly accessible TCGA data, are currently released through public portals, including the TCGA data portal [20], cBIO [21] and the University of California, Santa Cruz cancer genome browser [22]; data analysis results can also be directly downloaded from FIREHOSE, hosted by the Broad Institute [23] or the Sage Bionetworks Synapse repository [24]. Furthermore, data analysis can be performed using a number of tools including the portal’s GUI interfaces or sophisticated R packages such as TCGABioLinks [25]. The depth and breadth of TCGA protected sequencing data are ideal for bioinformatics methods development in novel areas such as RNA Seq variant calling pipelines, alignment free algorithms for mapping sequence data, development of predictive cancer biomarkers and continued improvement of variant calling pipelines. Access to protected TCGA data is made available through cgHUB [26] and only with a Data Use Certificate (DUC) from the database of Genotypes and Phenotypes (dbGAP).

While public portals provide initial access to the TCGA data and to a GUI interface for analysis results, the lack of a common data model, lack of interoperability between portals, and lack of programmatic access to the millions of data files produces significant limitations. For example, it is not uncommon for an identical query to yield different results from each of the portals. Investigators wishing to access TCGA data through public portals face technical challenges in navigating the various platforms, data types, data updates, file formats, and data levels. In addition, the sheer volume of data, absence of versioning, inconsistencies between analysis results, and inability to track changes in data files compound the difficulty of using data obtained from these portals in a reproducible fashion. Finally, for those investigators who need protected data files to develop new bioinformatics methods, even with a DUC, the typical investigator will not have the computing infrastructure required to download, store, manage, and analyze raw protected sequence alignment BAM files.

To address the informatics challenges in navigating the TCGA open access data, Robbins et al. developed the TCGA roadmap [27], which provides a data model and system for capturing, indexing, and annotating metadata for a subset of TCGA data. Metadata are represented in the Resource Description Framework (RDF), and queries leverage the SPARQL query language [28]. One of the key objectives of the TCGA roadmap project was to develop a programmatic interface that enables the end-user to navigate and discover subsets of files based on file-level provenance annotations and rich metadata. This enables, for example, discovery of the exact set of files that led to a particular research result, even if each patient sample, experiment, and platform has several raw data files (as a result of analysis pipeline updates). Although an important first step, the TCGA Roadmap is restricted to a small subset of TCGA datatypes and does not provide a direct way to version, archive, or harmonize the data.

Inspired by TCGA Roadmap [27] we developed TCGA Expedition to help advance our institutional capabilities at the University of Pittsburgh (Pitt) in NGS analysis and to support the research community focused on computational methods for cancer research. The resulting local repository, PGRR, serves the needs of more than fifty collaborating Pitt faculty members who are listed together on a single dbGAP DUC. Data obtained, versioned, and archived through the PGRR supports command line analysis at high-performance computing (HPC) facilities and also with third party tools. For a subset of TCGA data that were collected at Pitt/UPMC (partnering health care system), we also enrich the sparse TCGA clinical data with subsequent de-identified data from the electronic health record. Significant and unique advantages of PGRR over existing TCGA tools include the creation of a collaborative and common infrastructure of hardware and software for protected and public TCGA data and for a large number of investigators with diverse scientific objectives and different levels of bioinformatics skills. Here we describe the TCGA Expedition software as well as PGRR architecture, methods for loading of TCGA data, and security and regulatory controls that conform to new dbGAP best practices.

## Implementation

The TCGA Expedition software, which is open source and can be freely downloaded, consists of a set of scripts written in Bash, Python and Java. Like the TCGA Roadmap, our software downloads, extracts, harmonizes and stores TCGA metadata, which are then available for user query. Unlike TCGA Roadmap, our software also provides the capability to download and version all TCGA data (in addition to the metadata) by recursively traversing data files in each archive and identifying new and changed file versions. Scripts download each file independently and perform necessary validation routines. Files are split, parsed, and then renamed to increment the version in the current TCGA Expedition archive. The TCGA Expedition software generates a versioned, participant- and sample-centered, local TCGA data directory with metadata structures that directly reference the local data files as well as the original data files. Both RDF and relational data stores are available for the resulting TCGA metadata and support flexible searches of the data via a web portal (e.g., generate file manifest using metadata filters), user-centric data tracking tools (e.g., email notifications as files are changed or added), and data provenance tools (e.g., create data snapshot by date).

### Download Scripts and Data Validation

Download scripts, written in Bash, Java, or Python for a UNIX operating system, are used to securely obtain the latest version of all TCGA data types and store them to a participant/sample-centered repository; to create a repository, users specify the download directory, choose the database (or RDF store), and enter all usernames and passwords for TCGA and database access. The download scripts archive or delete previous versions and create or update metadata about downloaded files. Download scripts are separated into modules that access all four TCGA datastores (Table 1), including cgHub (BAM files only), firebrowse.org (level 4 Copy Number only), Georgetown (mass spectrometry data only), and TCGA (all other data types). TCGA download scripts utilize a configuration file to select datatypes. The user can select the frequency of interval downloads for all modules. In all cases, scripts check for new, modified, and deleted files by comparing downloads with existing files in the local repository. TCGA and firebrowse.org scripts compare the latest source archive number with the archive number in PGRR repository. cgHub scripts and scripts that download mass spectrometry data search for new/updated files starting from the last download date. Because of the massive size of BAM files, we have written scripts to download files on demand using GeneTorrent 3.8.7. BAM files are stored as separate analysis types for a corresponding sample.

**Table 1:** TCGA Expedition Modules and associated TCGA Datatypes managed.

TCGA Datatype	Data Source	Level	File Type	Repository Code Module
WGS_(cgHub)	cgHub	1	Bam	python script + BAMMetadataManager
WXS_(cgHub)	cgHub	1	Bam	python script + BAMMetadataManager
Protected_Mutations	TCGA	2	Vcf	ProtectedMutationsNoSplit
Protected_Mutations_MAF	TCGA	2	Maf	MafModule
Somatic_Mutations	TCGA	2	Maf	MafModule
RNA-Seq_(cgHub)	cgHub	1	bam, fastq	python script + BAMMetadataManager
RNASeq	TCGA	2	Vcf	RNASeqLevel2Module
RNASeq	TCGA	3	Txt	RNASeqLevel3Module
RNASeqV2	TCGA	3	Txt	RNASeqV2Level3Module
CNV_(CN_Array)	TCGA	1	txt, mat	CNAModule
	TCGA	2	Txt	CNAModule
	TCGA	3	Txt	CNAModule
CNV_(SNP_Array)	TCGA	1	txt, cel	CNVModule
	TCGA	2	Txt	CNVModule
	TCGA	3	Txt	CNVModule
	Firebrowse	4	txt, pdf, png, hml, gistic, Rdata, bed, properties	CN_Level4
CNV_(Low_Pass_DNASeq)	TCGA	2	Vcf	ProtectedMutationsNoSplit
Expression_Exon	TCGA	1	txt, cel	ExpExonModule
	TCGA	2	Txt	ExpExonModule
	TCGA	3	Txt	ExpExonModule
Expression_Gene	TCGA	1	txt, cel	ExpGeneModule
	TCGA	2	Txt	ExpGeneModule
	TCGA	3	Txt	ExpGeneModule
Expression_Protein	TCGA	0	Txt	ExpProteinModule
	TCGA	1	tif, txt	ExpProteinModule
	TCGA	2	Txt	ExpProteinModule
	TCGA	3	Txt	ExpProteinModule
	Georgetown	4	tar.gz, tsv	MassSpecModule
Bisulfite-Seq_(cgHub)	cgHub	1	Bam	python script + BAMMetadataManager
DNA_Methylation (array based)	TCGA	1	idat, txt	MethylModule
	TCGA	2	Txt	MethylModule
	TCGA	3	Txt	MethylModule
miRNA-Seq_(cgHub)	cgHub	1	Bam	python script + BAMMetadataManager
miRNASeq	TCGA	3	Txt	miRNASeqModule
Fragment_Analysis_Result (microsatellite instability)	TCGA	1	txt, fsa	MSIModule
Diagnostic_images	TCGA	1	Svs	ImageModule
Tissue_images	TCGA	1	Svs	ImageModule
Clinical (patient history and biospecimen)	TCGA	2	Txt	ClinicalModule

Data validation routines include: (1) checking analysis files to assure that TCGA barcodes correspond to analysis-specific universally unique identifiers (e.g. sample, aliquot, shipped portion) and correcting errors as needed; (2) standardizing tissue source site names for consistency using a manually created mapping file that accounts for alternative names, hyphens, misspelling, and extra spaces; (3) sorting variant call format (VCF) files by chromosome/position when they are not already sorted; (4) calculating MD5 checksums for BAM files and repeating downloads that do not generate the appropriate checksum; and (5) logging all downloads and identifying failed attempts for retry.

VCF data harmonization is accomplished by processing VCF headers and data using scripts written in Java. In TCGA, the VCF file structure varies from one analysis center to another (different header information, number of samples per file, number of files per sample, number of variant-callers used, etc.). We created a standard for the VCF file header that contains fileformat, filedate, center, platform, genome ref.name, genome ref.url, patient_id, and specimen_id followed by any additional information from the original data file. We also ensure that the reference positions (POS) are sorted numerically within each chromosome (CHROM) sequence.

### Versioning of files

TCGA Expedition code determines when a new version of a given file is made available through any of the TCGA associated data stores. Scripts traverse the public and controlled TCGA HTTP directories and download datasets from the latest experiments if (1) there no such data in the local TCGA Expedition repository or (2) the data have been modified within a subsequent archive version. When the data are reanalyzed, the archive version number changes, and the file CHANGES_DCC.txt is created, which lists all added, modified, or deleted analysis files. By parsing CHANGES_DCC.txt, our code determines when a newer version is available. If the analysis file is unchanged, we change the referenced archive number in the metadata. Otherwise, the file will be updated and marked according to the CHANGES_DCC.txt.

### Metadata management

TCGA metadata extraction scripts scrape essential metadata from various TCGA public sources, such as metadata available from TCGA reports [29]. Leveraging predicates extended from Robbins et al [27] we construct a metadata store that includes Disease Study, Tissue Source Site, Center, Sample Type, and Portion Analyte. These data are used to create description fields for the analysis file metadata. We also extract refGenome and refGenomeSource from mage-tab files. Metadata for algorithmName and algorithVersion are primarily parsed from the actual data files (for VCF) or are manually added (for DNAMethylation data). TCGA Expedition offers two options for storing metadata: either as RDF or in relational format.

#### Data files, naming conventions, and directory structure

**Fig 1:**
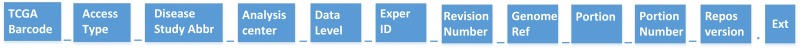
TCGA Expedition file name components.

For datatypes obtained from TCGA (Table 1), we extend the TCGA barcode to create more informative file names (Fig 1). TCGA Expedition file names begin with the (1) TCGA Barcode, followed by one or more of the following: (2) access type (public or controlled), (3) disease study abbreviation, (4) analysis center, (5) analysis platform, (6) TCGA data level, (7) experiment id from the TCGA archive name, (8) current analysis revision number for the TCGA experiment id, (9) TCGA revision number to distinguish between same type of files coming from different bulks for the same analysis type, (10) reference genome if available, (11) portion name to distinguish between files with the same extension and level but different types (e.g..idat files for DNA_Methylation have "red" and "grn" portions), (12) portion number to distinguish between files with the same extension, level, and the same type (the default value is "1"), and/or (13) TCGA Expedition Repository version number (incremented as modified files are downloaded).

Files downloaded from sources other than TCGA maintain their original names. For mass spectrometry data, we attach the analysis date prefix to the file names for convenience, since there is no versioning available.

**Fig 2:**
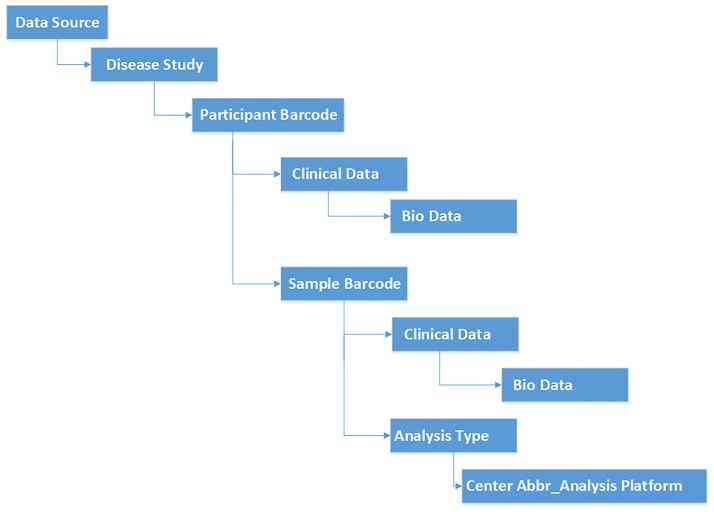
TCGA Expedition Directory Structure.

TCGA Expedition code modules create a simple participant-sample oriented directory based on metadata that is used to organize all downloaded data files. The hierarchy created is shown in Fig 2.

### Intranet Portal

**Fig 3:**
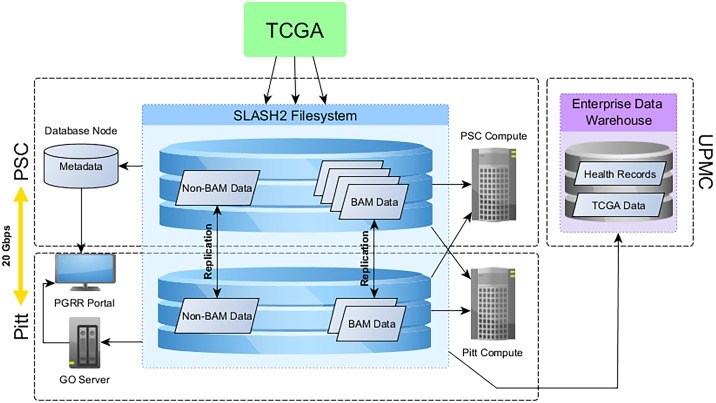
Architecture of the Pittsburgh Genome Resource Repository.

We provide an intranet portal for investigators to access files and to request notification of file changes. We developed the intranet portal using the Drupal content management system, REST-based application programming interface, structured query language queries to the PGRR database, and SPARQL queries to the RDF datastore. The portal provides the functionality needed to identify files of interest based on metadata query and filtering and to download a manifest of files and directory locations. The manifest can be easily leveraged in user-authored command line scripts for further data processing. The portal also provides notification mechanisms based on our RDF graph or relational metadata store (Fig 3). Investigators can identify when new samples are available and can request notification by email when a new version of a dataset of interest has been created at the PGRR. Thus, if a participant is deleted from the TCGA dataset at NCI, if a new set of samples appears, and/or if a file has been updated with meaningful differences, the resulting changes in the PGRR metadata repository are immediately communicated, and the investigator can take appropriate action, including re-analysis. Finally, the portal provides a way for investigators to request and maintain records of access for postdocs, staff, and students who are not required to be listed on dbGAP DUC.

### Systems architecture

PGRR was designed to facilitate the development of pipelines for analyzing NGS data, to support large-scale profiling and integrative analysis of genomic and phenotypic data, and to make these analytical resources accessible to both data scientists and investigators with little computational experience. To achieve these diverse goals, PGRR leverages two existing institutional HPC resources with complementary capabilities: the Pittsburgh Supercomputing Center (PSC) and the University of Pittsburgh Center for Simulation and Modeling (SaM).

Established in 1986, PSC is a joint effort of Carnegie Mellon University and the University of Pittsburgh and provides researchers nationwide with access to systems for high-performance computing, networking, and data-handling. SaM was created in 2008 to foster multidisciplinary research across the University of Pittsburgh and provide access to high-performance computing resources. Pitt is connected at network bandwidth of 100 Gb/sec through the Three Rivers Optical Exchange (3ROX) to PSC, Internet 2, and other research and education networks.

PSC storage resources dedicated to the PGRR are connected at a rate of 20 Gbps to the University of Pittsburgh via 3ROX. Pitt has also recently developed a Science DMZ which is configured with appropriate security controls for allowing movement of research data to HPC clusters and related data services without having to traverse enterprise firewalls. PGRR data transfer nodes at Pitt use the ScienceDMZ for un-firewalled paths to PSC.

The overall architecture of PGRR is shown in Fig 3 and encompasses storage and compute nodes, as well as metadata and application servers.

Storage resources provided through the PSC Data Exacell (DXC) system [30] make PGRR data accessible to both SaM and PSC computing systems through a SLASH2 wide-area filesystem (see below). Researchers with little computational experience can analyze the TCGA data through commercial applications such as GenomOncology and CLCBio; those with more computational experience can analyze the data using command-line tools on SaM or DXC computational systems at PSC. Access to SaM allows Pitt researchers to utilize their existing analysis frameworks that are already configured and supported, while access to PSC enables large-scale analyses, such as those requiring large shared memory (e.g. structural variation analysis or de novo assembly) or analyses across the entire TCGA dataset (including all BAM files).

### Data storage

At the core of PGRR is 1.1 PB of storage mounted as a wide-area SLASH2 filesystem [31] across storage resources at both SaM and PSC, which presents a single view of PGRR data to researchers, while still allowing multiple transparent “replicas” of the data to be accessible locally from SaM, PSC, or both. This transparency is possible through the replication engine of the PSC-developed SLASH2 filesystem [32], which creates and manages multiple copies of data at the file-chunk level and automatically provides access to the closest replica of a requested piece of data, thus maximizing performance. Despite the distributed nature of the data, researchers see only a single copy of the data; the paths used to access the data never change. This is true regardless of where or how researchers access the data and makes it easy to track changes in the data and ensure that all data are up to date. This method also enhances security, because researchers can work “read-only” with the protected TCGA data, and only write out secondary analyses to other filesystems.

To facilitate movement of replicated data between SaM and PSC, we have integrated a data scheduling utility, called DMOVER (publically available) [33], into our infrastructure. DMOVER orchestrates and verifies SLASH2 data transfers in cases where local copies are required. For example, if researchers using SaM need to have a local replica of the data for performance or other reasons, they can schedule a data replication job as a dependency to their compute job. The compute job will not execute until all requested data sets have been replicated to SaM resources.

### Loading TCGA data to other systems

Once files are downloaded, validated, and processed using TCGA Expedition, they can also be loaded into appropriate viewing and analysis software. Both open source tools such as cBIO [34] and commercial software such as CLC Bio [35] and Oracle Translational Research Center [36]; have been utilized for visualization and analysis of TCGA data. To simplify the process of identifying when new and modified files are available, TCGA Expedition includes scripts for generating JSON messages with each set of downloads. In our environment, JSON messages [37] are used for loading data to separate software systems. The same code could be repurposed by others to schedule extract, transform, and load processes of TCGA data into other downstream platforms.

We provide two methods for the visualization and analysis of TCGA data. First, we licensed code from GenomOncology software [38] developed by a regional start-up that had already worked with TCGA data. The company modified GenomOncology specifically for our project, including restricting the ability of users to download any germline sequence data.

Second, for UPMC patients whose consent permits re-association and incorporation of additional data from their electronic health record, PGRR orchestrates the copy and movement of TCGA files derived from these UPMC patients into the UPMC Enterprise Analytics Data Warehouse [an in-house Oracle data warehouse, using tools from the Oracle Translational Research Center (TRC)]. TCGA files from NGS platforms can be loaded by script into the TRC Omics Data Bank, a rich relational model for NGS data; phenotype data derived from electronic health record are associated with the NGS data within the Cohort Datamart. TRC also includes additional tools such as the Clinical Development Center, the Oracle Cohort Explorer, and Oracle R. We are also currently developing scripts for loading TCGA data to tranSMART [39].

### Regulatory Compliance

Faculty users of TCGA data hosted through the PGRR are co-investigators on a dbGAP DUC, and research by these investigators is limited to the description listed on the approved DUC. At Pitt, the development and implementation of the PGRR has been approved by the University of Pittsburgh Institutional Review Board (PRO12090374). The hosting of these data by PSC and UPMC is covered under separate service agreements.

Re-associated TCGA-electronic health record data (described above) is provided back to researchers as de-identified data only. To date, this process has been performed with 139 consented breast cancer patients from UPMC whose tumor samples and limited de-identified clinical data were contributed to TCGA.

### Data Security

The security measures in place to protect TCGA data being stored and analyzed in the PGRR have undergone a formal review process involving security officers and technical staff at Pitt and PSC to ensure they are aligned with National Institutes of Health security best practices. Nodes in the Pitt Science DMZ are protected using router Access Control Lists (ACLs). All other cluster nodes at SaM are secured behind the University firewall, which allows only internal access or access through the University virtual private network. In both cases, only encrypted access is allowed (e.g., using SSH). All extraneous services are controlled at the firewall (e.g., email, file sharing, printing, etc.). All user accounts are password-restricted, with strong password policies dictating the content of passwords and requiring password rotation. All data on the cluster are restricted using filesystem ACLs so that only appropriate project members have access to project data. These measures are also observed at PSC. SLASH2 supports UID and GID mapping so individual and group permissions that control access to protected data at Pitt are propagated to PSC such that only authorized Pitt users can access protected data at PSC. Communication between nodes at Pitt and PSC are enabled through implementation of specific firewall rules (or router ACLs in the case of DMZ nodes) that enable access between those machines. The Enterprise Analytics Data Warehouse is positioned behind the UPMC firewall, is subject to UPMC security scans, and meets all dbGaP security requirements.

## Results and Discussion

Leveraging the TCGA Expedition codebase, the PGRR has been in operation at the University of Pittsburgh for nearly two years. The deployed system currently includes many TCGA datatypes in their entirety and at least some of every data type currently available. A total of 75 active users include 18 collaborating faculty along with their trainees and staff.

To help investigators at our institution work with large NGS data such as TCGA, we provide multiple levels of support including, 1) direct command line access, compute allocation and storage through our partners at SaM and PSC, 2) technical help desk support through SaM and PSC, 3) for end-users without bioinformatics training, analytic support through the CBS, a data analysis core at the University of Pittsburgh Cancer Institute, and 4) monthly introductory training sessions that explain the PGRR infrastructure, types of data, requirements for accessing the data, and the various resources available to assist them. Additionally, we hold an annual Pittsburgh TCGA Community Meeting that allows investigators to provide input on useful modifications and additions to the infrastructure; this forum also provides a chance for diverse investigators to share progress on their scientific goals and accomplishments.

PGRR’s broad utility across institution is possible through the open source file and metadata management codes which can easily be deployed by institutions with adequate hardware and IT infrastructure to support such Big Data. Beyond its inter-institutional utility, PGRR has demonstrated significant advantage over public TCGA portals for the University of Pittsburgh TCGA investigators for whom PGRR provides a centralized repository of harmonized and versioned data. These investigators have multiple mechanisms to access the data including through command line interface, through third party GUI tools or by utilizing the services of the bioinformatics core.

### Use cases

In PGRR’s two years of operation with access by dozens of users, a number of use cases have emerged which highlight the utility of PGRR for University of Pittsburgh cancer research groups. Some of the use cases include 1) understanding the effect of variant callers on final list of validated variants; variant calling pipelines differ based on platforms, sequencing centers, and reference genome version used. The TCGA barcodes do not provide sufficient enough information to extract this information without detailed investigation of the metadata files which can be prohibitively difficult when working with a large number of files. The human readable PGRR file names which contains detailed harmonized metadata such as institution, platform, analyte, and variant caller, has enabled the CBS group to easily subset data by various parameters and to analyze the effect of these parameters on final list of variants. PGRR data harmonization ensures that inconsistencies such as different names for the same institution or unavailability of analytical pipeline names in public portals are, in fact, resolved in the PGRR metadata repository, 2) data versioning and the snapshot feature of the search repository has enabled data freezes so that analysis results are unaffected and can be compared to deprecated pipelined and also TCGA data updates, 3) detailed analysis of the protected Level 2.vcf files to examine the sequencing depth and quality of variants between normal and tumor samples which allows investigators to filter variants by their own criteria and compare results to publicly available MAF files. This analysis both via command line and GUI tools such as GO has been particularly useful for comparing sequencing results of samples submitted by the University of Pittsburgh to in-house sequencing data from these same samples, 4) annotation of level 2 vcf files rather than publicly available list of variants has allowed investigators to utilize their own custom criteria for filtering putative somatic variants, 5) lncRNA analysis by one group of investigators who re-aligned 1098 breast cancer FASTQ files to GTF files that contain lncRNAs, resulting in new BAM files and quantification of lncRNA expression. Some of these researchers have begun to test and compare multiple RNA quantification algorithms, including TopHat/cufflinks and STAR/HTSeq. Similarly, access to the FASTQ files has made it possible to test new alignment-free quantification tools such as SailFish. The above mentioned examples of PGRR utility for a large group of investigators was achieved by both large-scale (e.g., thousands) processing and analysis of relatively small data files (e.g., 1–5 GB RNA-seq BAM file) and small-scale processing and analysis of large data files (e.g. 100 Gb WGS BAM files).

### Institutional capability and enhancing collaboration

The creation of the PGRR has also helped advance institutional capabilities, including the improvement of infrastructure for managing NGS data. Investigators with limited prior experience have become more comfortable with these new data formats, analysis pipelines, and associated tools. WGS data are especially challenging for individual investigators to use, due to issues with downloading, storage, and processing. Having WGS BAM files all located as a local resource enables investigators to examine the importance of somatic non-coding mutations in cancer. For example, investigators have used the breast cancer WGS BAM files to examine non-coding mutations in transcription factor binding sites in enhancer regions many Kb away from a gene, and validated mutations that affect transcription factor binding and subsequent gene expression (manuscript in preparation).

Additionally, PGRR infrastructure has been an important resource for large data-centric collaborations and research centers, such as the Center for Causal Discovery (U54HG), a Big Data to Knowledge Center of Excellence that is developing and making available state-of-the-art algorithms for causal discovery and modeling, including the analysis of TCGA data for driver mutations and combinations of mutations [40]. The Big Data for Better Health is another big data initiative, funded by the Pennsylvania Department of Health, which uses machine learning methods for prediction in cancer.

We anticipate building on the PGRR infrastructure to support the use of other large-scale datasets, such as the Framingham heart study [41]. Extensions to other datasets may require modifications of our download code but can maintain the same metadata repository, directory structure, portal, and JSON messaging approaches.

## Conclusions

We describe our TCGA Expedition software for acquiring and managing TCGA data, our implementation of the software to create a local collaborative infrastructure, and the impact of that infrastructure on our cancer research programs. TCGA Expedition is open source and freely available. Collaborative groups and institutions can emulate our approach to create enhanced local capabilities for addressing many of the challenges of working with these datasets, particularly the use of a centrally managed infrastructure for research NGS data to ensure consistency and reproducibility.
